# Spontaneous regression of micro-metastases following primary tumor excision: a critical role for primary tumor secretome

**DOI:** 10.1186/s12915-020-00893-2

**Published:** 2020-11-06

**Authors:** Lee Shaashua, Anabel Eckerling, Boaz Israeli, Gali Yanovich, Ella Rosenne, Suzana Fichman-Horn, Ido Ben Zvi, Liat Sorski, Rita Haldar, Ronit Satchi-Fainaro, Tamar Geiger, Erica K. Sloan, Shamgar Ben-Eliyahu

**Affiliations:** 1 Sagol School of Neuroscience and School of Psychological Sciences, Tel Aviv University, 69978 Tel Aviv, Israel; 2 Department of Human Molecular Genetics and Biochemistry, Sackler Faculty of Medicine, Tel Aviv University, Tel Aviv, Israel; 3 Pathology Institute, Rabin Medical Center, Tel Aviv University, Petach Tikva, Israel; 4 Neurosurgery Department, Rabin Medical Center, Tel Aviv University, Petach Tikva, Israel; 5 Department of Physiology and Pharmacology, Sackler Faculty of Medicine, Tel Aviv University, Tel Aviv, Israel; 6 Drug Discovery Biology Theme, Monash Institute of Pharmaceutical Sciences, Monash University, Parkville, VIC 3052 Australia

**Keywords:** Metastatic regression, Breast cancer, Surgery, Removal of primary tumor, Cancer secretome

## Abstract

**Background:**

Numerous case studies have reported spontaneous regression of recognized metastases following primary tumor excision, but underlying mechanisms are elusive. Here, we present a model of regression and latency of metastases following primary tumor excision and identify potential underlying mechanisms.

**Results:**

Using MDA-MB-231^HM^ human breast cancer cells that express highly sensitive luciferase, we monitored early development stages of spontaneous metastases in BALB/c nu/nu mice. Removal of the primary tumor caused marked regression of micro-metastases, but not of larger metastases, and in vivo supplementation of tumor secretome diminished this regression, suggesting that primary tumor-secreted factors promote early metastatic growth. Correspondingly, MDA-MB-231^HM^-conditioned medium increased in vitro tumor proliferation and adhesion and reduced apoptosis*.* To identify specific mediating factors, cytokine array and proteomic analysis of MDA-MB-231^HM^ secretome were conducted. The results identified significant enrichment of angiogenesis, growth factor binding and activity, focal adhesion, and metalloprotease and apoptosis regulation processes. Neutralization of MDA-MB-231^HM^-secreted key mediators of these processes, IL-8, PDGF-AA, Serpin E1 (PAI-1), and MIF, each antagonized secretome-induced proliferation. Moreover, their in vivo simultaneous blockade in the presence of the primary tumor arrested the development of micro-metastases. Interestingly, in the METABRIC cohort of breast cancer patients, elevated expression of Serpin E1, IL-8, or the four factors combined predicted poor survival.

**Conclusions:**

These results demonstrate regression and latency of micro-metastases following primary tumor excision and a crucial role for primary tumor secretome in promoting early metastatic growth in MDA-MB-231^HM^ xenografts. If generalized, such findings can suggest novel approaches to control micro-metastases and minimal residual disease.

## Background

Surgical removal of the primary tumor is a cornerstone of cancer treatment. Nevertheless, this life-saving approach has been suggested to accelerate the post-operative progression of minimal residual disease through processes triggered by the surgical procedure itself [1, 2] or by the elimination of inhibitory signaling from the primary tumor [3].

However, various case studies report the opposite effect: spontaneous post-operative regression of evident cancer metastases or malignant foci [4–8]. Such post-operative regression, although rare, has been clearly documented in most types of cancer [8], including breast cancer [9], and has been most commonly reported for lung metastases [8–14].

Several underlying mechanisms have been suggested to elicit spontaneous post-operative regression of residual malignant foci [12, 15, 16], including surgical trauma [5], and elimination of stimulating factors secreted by the primary tumor or induced by its presence [16]. Unfortunately, postulated mechanisms have not been empirically tested, as no animal model of such spontaneous regression exists. The need to study potential interactions between the primary tumor (PT) and metastases is stressed by the current realization that the presence of a primary tumor is a systemic disease and that a continuous crosstalk between the PT, its microenvironment, and distant organs plays a significant role in disease etiology and progression [17, 18].

Herein, for the first time, we present an animal model of spontaneous regression of metastases following PT removal. This model employs a highly sensitive luciferase reporter of cancer cells, which enables the study of early-stage micro-metastases. We found that the development of micro-metastases is supported by the numerous factors secreted from the PT and that removal of the PT and its secreted factors induces the regression of early-stage metastases. Specific potential factors were identified, and an in vivo neutralization of four of them in the presence of the PT halted the progression of metastases.

## Results

**Fig. 1:**
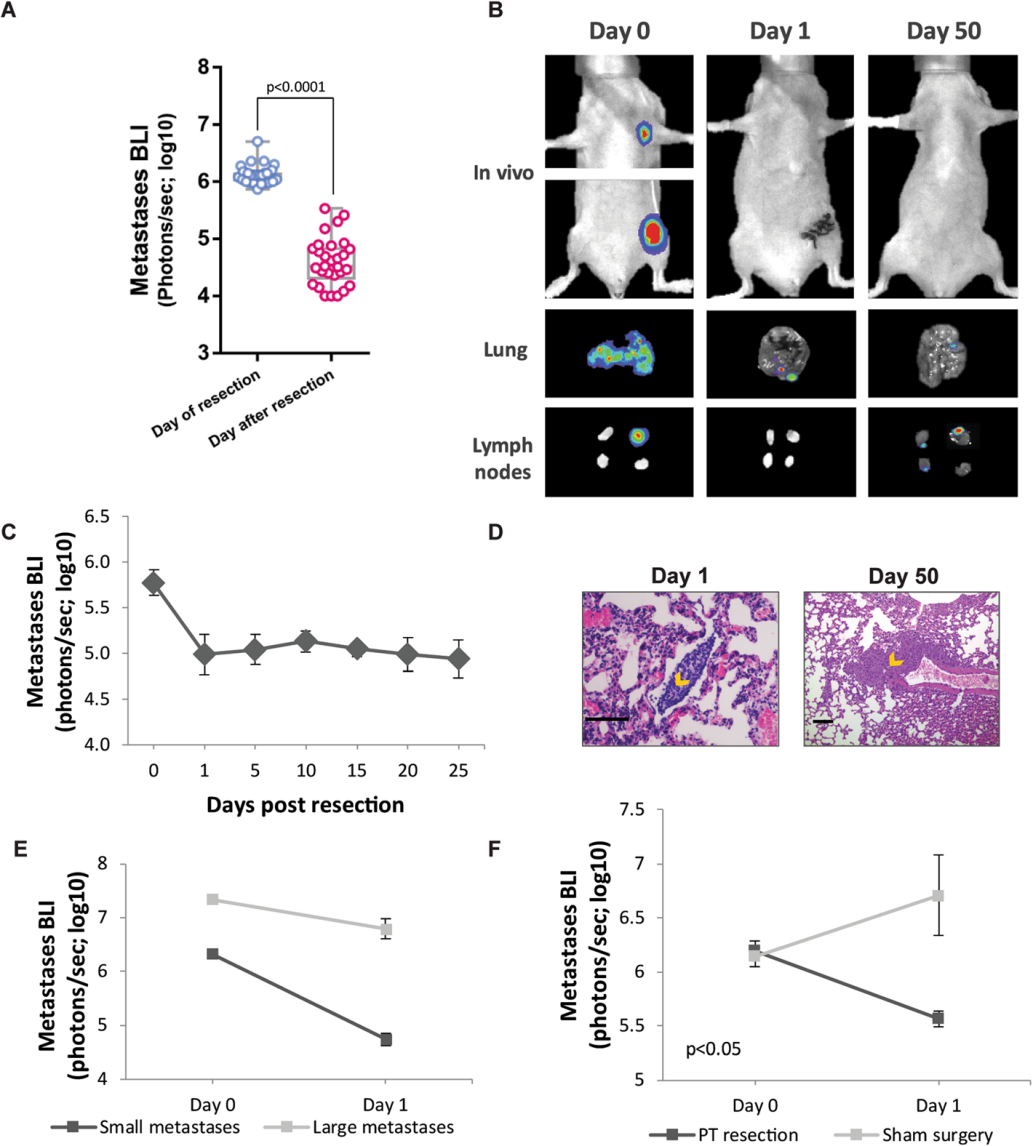
Excision of the primary tumor elicits regression of early-stage metastases. **a** In vivo quantification of lung and lymph node metastasis by bioluminescence imaging (BLI) immediately before (day 0) and after primary tumor (PT) resection (day 1) (*n* = 29). Whiskers represent the min and max points. **b** Representative images of lung and lymph node (LN) metastases, in vivo and ex vivo at day 0 and post-operative days 1 and 50, following tumor excision. **c** In vivo BLI of metastases over time (*n* = 6). **d** Representative images of H&E staining of the lung sections at post-operative days 1 and 50. Orange arrows indicate micro-metastases. Scale bar day 1 = 100 μm, scale bar day 50 = 225 μm. **e** In vivo quantification of lung and LN metastases by BLI before (day 0) and after PT resection (day 1) in mice bearing small (*n* = 7) or large (*n* = 9) metastases. **f** In vivo quantification of lung and LN metastases by BLI before (day 0) and after (day 1) sham surgery or PT resection. The detection threshold for all BLI figures is ~ 10^4^ photons/s (i.e., log_10_ = ~ 4). Error bars in **c**, **e**, and **f** represent mean ± SE

To examine the effect of primary tumor excision on metastatic growth, MDA-MB-231^HM^ cells were injected into the mammary fat pad of nude mice to form a primary tumor. In this orthotopic model, distant metastases are formed spontaneously in the lymph nodes and lungs 2–4 weeks following tumor implantation. When the chest-localized bioluminescent signal of metastases reached a total flux of 10^6^ photons/s, primary tumors were excised. A dramatic decrease in metastatic signal, of up to 100-fold, was evident in mice along the 24 h following tumor excision, *p* < 0.0001 (Fig. 1a, b). This decrease occurred gradually along the first 24 h, and seemed to continue for another 1–2 days thereafter, as evident in Additional file 1: Fig. S1. These metastases have regressed into latent foci, as (i) no increase in in vivo bioluminescent signal occurred for 50 days post-excision (Fig. 1b, c) and as (ii) microscopic malignant foci were evident on both the day after tumor excision (day 1) and 50 days later, as confirmed by H&E staining of the lungs and lymph nodes (Fig. 1d). To test whether regression is specific to early metastatic stages, we then excised tumors from mice bearing either small (10^6^ photons/s) or large (10^7^ photons/s) metastases and found a significantly less prominent regression in larger metastases, *p* < 0.0001 (Fig. 1e).

As several mechanisms have been proposed to explain spontaneous regression, we first sought to distinguish between the effects elicited by the surgical procedure itself (such as inflammation and vascular insufficiency) and those that are related to the removal of the tumor mass, including the elimination of primary tumor-derived pro-metastatic secreted factors (i.e., growth factors, cytokines, and angiogenic factors). To this end, mice underwent either sham surgery (sparing the primary tumor) or primary tumor excision. Metastases regressed significantly following excision of the primary tumor, while continued to increase in mice subjected to sham surgery, *p* < 0.05 (Fig. 1f).

**Fig. 2:**
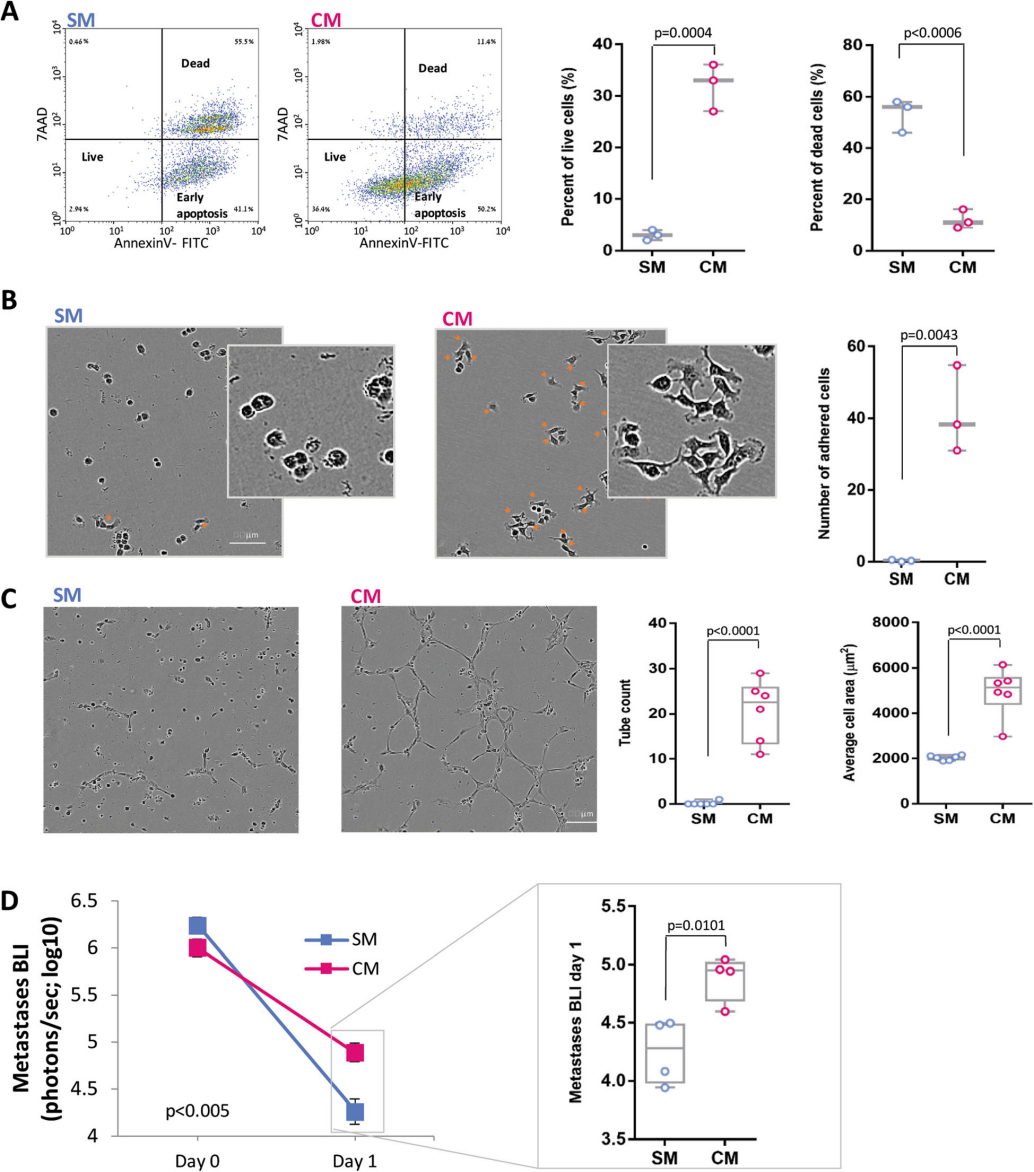
CM effects on pro-metastatic processes and metastasis. **a** Representative images and quantification of flow cytometry for AnnexinV and 7AAD of MDA-MB-231^HM^ cells that were grown in serum-free medium (SM) or MDA-MB-231^HM^ tumor cell-conditioned medium (CM). **b** Representative images and quantification of adhered cancer cells incubated with SM or CM. Orange arrows mark adhered cells. Scale bar, 100 μm. **c** Representative images and quantification of tube formation by human endothelial cells incubated with SM or CM on a layer of basement membrane extracellular matrix. Scale bar, 100 μm. **d** In vivo quantification of lung and LN metastases by BLI before (day 0) and after PT resection (day 1) in mice that received SM or CM, simultaneously with tumor excision (*n* = 4 per group). The detection threshold is ~ 10^4^ photons/s (i.e., log_10_ = ~ 4). Whiskers in **c** and **d** represent the min and max points. Error bars in **a**–**d** represent mean ± SE

As the spontaneous regression was elicited by the removal of the primary tumor, we hypothesized that the primary tumor secretome supports the survival and growth of distant micro-metastases, and thus, its elimination by surgery results in metastatic regression. As regression was more prominent in small metastatic foci (Fig. 1e), we investigated events that characterize the early stages of metastasis development. In order to simulate early-stage micro-metastases and study the contribution of tumor secretome to their development, cells were seeded in vitro in low numbers and density (~ 10% confluence). We found that conditioned medium by MDA-MB-231^HM^ tumor cells, containing tumor-derived secreted factors (compared to serum-free medium), enhanced viability (*p* < 0.001; Fig. 2a) and adhesion (*p* < 0.01; Fig. 2b) of these sparse cancer cells and induced tube formation of human endothelial cells (*p* < 0.0001; Fig. 2c). To study the in vivo effect of secreted factors on metastasis, osmotic mini-pumps containing tumor cell-conditioned medium (vs. serum-free medium) were implanted at the time of tumor excision to partly replenish the primary tumor secretome. Mice that were treated with tumor cell-conditioned medium showed 10-fold less regression than mice that received serum-free medium, *p* < 0.005 (Fig. 2d), indicating that secreted factors from the primary tumor support survival of distant metastases. We estimate that the amount of secreted factors released daily by the osmotic mini-pumps was ~ ^1^/_8_ of the amount produced daily in vivo by the primary tumor (see the “Methods” section).

To identify factors whose elimination mediates the spontaneous regression of metastases following primary tumor excision, tumor secretome was analyzed employing a human cytokine array. We analyzed 4 conditions: (i) tumor cell-conditioned medium (CM) and plasma samples from (ii) non-tumor-bearing mice, (iii) primary tumor-bearing mice, and (iv) mice 1 day following tumor excision. We identified 28 cytokines that were highly expressed in cancer cell CM (normalized mean intensity > 6000). Out of those 28 cytokines, we chose factors that were highly expressed in the plasma of tumor-bearing mice (intensity > 2000) and, of these factors, selected those that were higher relative to their plasma levels in (i) non-tumor-bearing mice (ratio > 1.5) and (ii) in mice 1 day following tumor excision (ratio > 1.3) (see Additional file 1: Table S1). This selection method pointed at four factors that are also known to exert pro-metastatic or pro-survival activities, and thus, their elimination may induce spontaneous regression: interleukin-8 (IL-8), platelet-derived growth factor-aa (PDGF-AA), Serpin E1 (also known as plasminogen activator inhibitor-1 (PAI-1)), and macrophage migration inhibitory factor (MIF). The presence of these 4 factors in CM was validated using ELISA (Additional file 1: Fig. S2). A fifth factor, DKK1, which was also pointed out by the cytokine array data, was not studied herein, given the mixed literature reports regarding its impact on malignant progression [19–22].

**Fig. 3.**
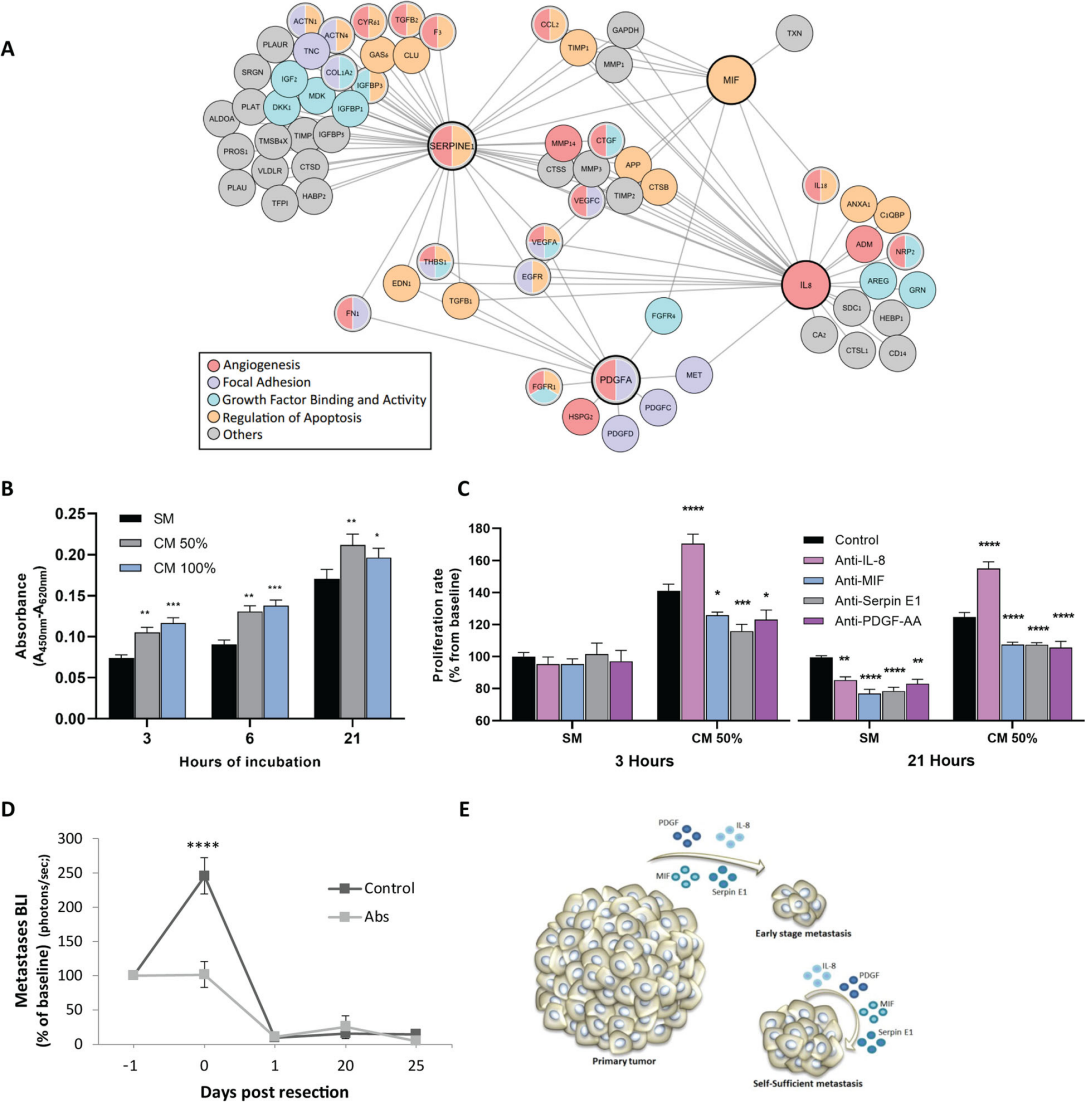
Secreted factors and pathways potentially underlying metastatic regression. **a** Proteomic and GO enrichment analysis identified a network of proteins and enriched pathways. Proteins in this scheme are those connected to at least one of the 4 key factors (denoted by a thick line). **b** Proliferation of MDA-MB-231^HM^ cells that were cultured in serum-free medium (SM) or in 100% or 50% MDA-MB-231^HM^ tumor cell-conditioned medium (CM), measured after 3, 6, or 21 h of incubation (3 h, *n* = 20 per media type; 6 and 21 h, *n* = 16 per media type). Asterisks represent a significant result relative to the SM group in each time point. **c** Proliferation of MDA-MB-231^HM^ cells that were cultured for 3 or 21 h in SM or CM 50% with IgG isotype control or antibodies against either IL-8, PDGF-AA, Serpin E1, or MIF. Data is presented as percent from IgG control SM in each time point (each antibody, *n* = 4; IgG control, *n* = 16). Asterisks represent a significant result relative to the control group. **d** In vivo long-term quantification of metastasis following cocktail administration of the four neutralizing antibodies (IL-8, MIF, PDGF-AA, and Serpin E1; *n* = 4) or IgG control (*n* = 5). **e** A scheme of the hypothesized model based on our results, suggesting that primary tumor secretome is crucial for the survival of early-stage micro-metastases, but not for larger metastases. Abs, antibodies. Error bars in **b**–**d** represent mean ± SE. ****p < 0.0001, ***p < 0.001, **p < 0.01, *p < 0.05

To complement this “narrow-down” approach, we used an unbiased mass spectrometry proteomic analysis of the CM, and identified 2600 proteins, 359 of which were annotated as extracellular factors by Gene Ontology (GO) analysis [23], including the four factors identified using the cytokine array (Additional file 2: Table S2–3). Pathway enrichment analysis of the 359 extracellular proteins identified proteins engaged in key steps of metastasis, including apoptosis, angiogenesis, growth factor activity, focal adhesion, and metalloenzyme regulation. Examination of protein-protein interaction networks using the String database identified pathways involved in the early stages of metastasis (Fig. 3a) [24].

In order to test the specific contribution of each of the four factors to MDA-MB-231^HM^ tumor cell survival and growth, we conducted a proliferation assay using WST-1 reagent (see the “Methods” section). First, we studied the dose- and time-dependent effects of MDA-MB-231^HM^-conditioned medium (CM) on cell proliferation. Tumor cells were seeded in low density (~ 10% confluence, 2500 cells, 100 μl/well) in CM 100%, CM 50% (diluted in serum-free medium (SM)), or SM (0% CM) for an incubation period of 3, 6, or 21 h. Two-way ANOVA and subsequent post hoc analyses revealed that proliferation rates were the lowest in SM compared to both CM 100% and CM 50%, in each of the three time points we tested (*p* < 0.05 for all comparisons) (Fig. 3b), supporting our hypothesis that CM promotes tumor cell growth. We then continued to test neutralization of proliferation for each of the four factors, comparing SM to CM 50% following 3 and 21 h of incubation, using mouse anti-human antibodies to either IL-8, PDGF-AA, Serpin E1, MIF, or IgG for control. The results indicated that except for IL-8 that showed mixed results, the blockade of each factor at 3 h reduced proliferation in CM 50%, but not in SM, and at 21 h reduced proliferation in both SM and CM 50% (*p* < 0.05 for all comparison) (Fig. 3c). We hypothesize that the antibody-dependent blockade of proliferation in the SM was effective at 21 h through the blockade of accumulated self-secretion of the 4 factors.

Next, we hypothesized that if these four factors are among those that drive metastatic growth, their in vivo blockade would partly mimic the effect of primary tumor removal and inhibit metastasis. To test this hypothesis, a cocktail of the 4 antibodies or IgG control was injected 24 h prior to tumor excision, at an early metastatic stage (chest bioluminescence < 10^6^ p/s). Antibody neutralization of these factors completely blocked the metastatic progression, *p* < 0.0001 (Fig. 3d). As could be expected, no difference in regression of metastases was evident between these two groups on post-excision day 1, likely as excision completely removed all secreted factors in both groups. These findings suggest that these primary tumor-secreted factors are among the factors crucial for survival and progression of early-stage micro-metastases, while established larger metastatic foci may be self-sufficient (Fig. 3e).

**Fig. 4.**
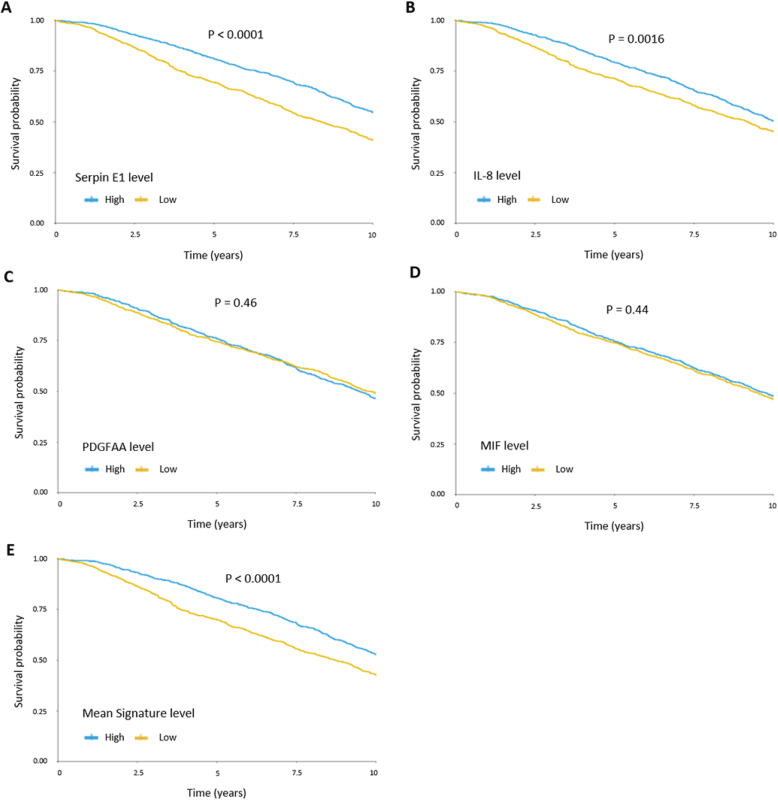
Associations between levels of Serpin E1, IL-8, MIF, and PDGF-AA and survival in breast cancer patients. The METABRIC dataset was used to assess the association between expression levels of Serpin E1 (**a**), IL-8 (**b**), PDGF-AA (**c**), MIF (**d**) and the mean signature levels of all 4 factors (median of the mean of normalized expression levels of the four factors) (**e**), with 10-year survival. Protein levels were classified as higher or lower than the median, and the association to 10-year survival was assessed by the Kaplan-Meier analysis (*n* = 952 per group). *p* value was calculated using two-sided log rank test

To start exploring the clinical relevance of these tumor-derived factors, we retrospectively studied patient outcome in the METABRIC breast cancer cohort, as well as TCGA lung adenocarcinoma dataset, as the lungs were the major site of metastasis in this study. In the breast cancer cohort, studying each factor alone, high levels of either Serpin E1 and IL-8 were associated with poor survival (*p* < 0.0001 and *p* = 0.0016, respectively), whereas high expression levels of MIF and PDGF-AA did not predict poor survival (Fig. 4a–d). In both the breast cancer cohort and the lung adenocarcinoma cohort, we found that high protein levels of all four factors (IL-8, PDGF-AA, Serpin E1, and MIF) were associated with significantly lower survival (*p* < 0.0001 and *p* < 0.05, respectively) (Fig. 4e, Additional file 1: Fig. S3). Additionally, we studied 4 more factors that were pointed out by the cytokine array in the current study (Additional file 1: Table S1), but did not pass all our selection criteria, and appeared in the METABRIC breast cancer database: DKK1, IL-6, LIF, and M-CSF. Of these four factors, high levels of IL-6 were associated with poor patient survival (*p* = 0.0005) (Additional file 1: Fig. S4).

## Discussion

The findings of this study indicate a crucial supportive role for MDA-MB-231^HM^ tumor secretome in survival and progression of distant metastatic foci and demonstrate the phenomena of metastatic regression and/or dormancy following primary MDA-MB-231^HM^ tumor removal. We identified four factors secreted by MDA-MB-231^HM^ cells, IL-8, MIF, Serpin E1, and PDGF-AA, which are known to affect malignant progression, and showed that (i) neutralization of each one of them, except IL-8 that showed mixed effects, decreased MDA-MB-231^HM^ proliferation in vitro and (ii) their combined in vivo neutralization in the presence of the PT arrested metastatic progression. Using the METABRIC database of breast cancer patients, we found that high tumor expression levels of either Serpin E1 or of IL-8 alone predict poor survival. High expression levels of all four factors predict poor survival in both the METABRIC cohort of breast cancer patients and the TCGA cohort of lung adenocarcinoma patients. These results may reflect the potential role of tumor secretome on metastatic growth and ultimately on patient long-term cancer outcomes. Our findings suggest that tumor-secreted factors may act through multiple mechanisms to affect both malignant cells and/or their microenvironment (Fig. 2). The model of regression of metastases presented herein presents an opportunity to empirically study metastatic regression into latent foci, and to further explore mediating mechanisms, with the goal of identifying novel prophylactic and therapeutic strategies.

It is well established that the primary tumor secretes numerous factors (e.g., growth factors, angiogenic factors, cytokines, exosomes, and hormones) that promote its growth, modulate its microenvironment [17], and induce a “pre-metastatic niche” at distant organs [18, 25]. Nonetheless, the presence of a primary tumor is also believed to be metastasis-inhibitory [26], through other mechanisms. The current study shows that primary tumor secretome can be a vital factor in promoting metastasis during the early stages of metastatic formation. This phenomenon may occur in various animal models and in a substantial portion of cancer patients, but remains unrecognized, given the limited detection capability of early micro-metastases, especially in the clinical setting. Here, we used high-sensitive codon-optimized luciferase-2 (luc-2) and focused on the earliest time of recognizable metastatic development, which enabled us to detect micro-metastases and their regression into latent foci in these early stages. Larger micro-metastases showed lower or no regression, potentially given their ability to secrete a sufficient amount of the necessary factors. This may also be the case in the great majority of cancer patients who exhibit detectable macro-metastases. Overall, regression of micro-metastases may be a common phenomenon in the clinical setting, but a condition that is currently unnoticeable and unappreciated clinically or therapeutically.

Several mechanisms were previously suggested to underlie spontaneous regression. As surgical procedure without primary tumor excision did not elicit any regression of metastases in the current study, the hypothesized mechanism of surgical trauma and its associated processes is negated in the current setting. Interestingly, several clinical studies reported spontaneous regression of metastases following radiation or cryotreatment of the primary tumor [27, 28]. These cases could be explained by the current results, as these treatments largely cease the secretion of most factors by the manipulated primary tumor. Also, radiation or cryotreatment is expected to induce lower levels of post-treatment systemic stress-inflammatory responses, compared to excision surgery [29]. These lower responses may reduce the known pro-metastatic effects of standard surgical procedures [1, 29, 30], increasing the anti-metastatic impact of the elimination of tumor secretome [31].

Our work proposes a role for IL-8, MIF, Serpin E1, and PDGF-AA in maintaining and promoting micro-metastases, through shared and distinct pathways. All four factors have been shown to promote angiogenesis and/or to suppress tumor apoptosis. Specifically, IL-8 is a pro-angiogenic and pro-inflammatory chemokine that was shown to promote invasion of tumor cells and enhance malignant survival [32–36]. Serpin E1 (PAI-1) was shown to promote metastases by (i) increasing thrombosis which supports angiogenesis [37–39] and (ii) inducing pro-survival and anti-apoptotic activities in tumor cells [40–42]. The proangiogenic factor, PDGF-AA, was shown to act as a survival factor to inhibit apoptosis [43] and to stimulate reorganization of actin [44]. The pro-inflammatory factor, MIF, was shown to promote metastases by (i) initiating the NF-κB signaling cascade resulting in the secretion of pro-inflammatory cytokines such as IL-8, TNF-α, IL-1, and IL-6; (ii) promoting MMPs activity; (iii) increasing tumor infiltration of myeloid-derived suppressor cells [45, 46]; (iv) promoting EMT [47]; and (v) exerting pro-survival [48] and anti-apoptotic activities [49]. Herein, we found that all four factors increased MDA-MB-231^HM^ proliferation in vitro, except IL-8 that showed mixed results at different CM concentrations. Overall, these PT-secreted factors are likely to promote metastatic progression in micro-metastatic niches, especially when the autocrine release of growth-supporting factors is insufficient.

Given the multiple factors potentially mediating the pro-metastatic effects of MDA-MB-231^HM^ secretome, we herein tested only four factors (of the 359 extracellular secreted factors) that we hypothesized to have the most significant impact. We tested and found separate in vitro effects for each of the four factors and showed that their combined in vivo blockade completely halted the progression of micro-metastases in the presence of the PT. Other secreted factors are likely to also be involved in supporting metastatic progression, including IL-6 that herein was upregulated in the plasma of tumor-bearing mice and that its tumor expression levels in the METABRIC database predict lower breast cancer patient survival. Different tumors, specifically syngeneic lines, should be similarly studied to test the generalizability of the novel phenomenon observed in the current study and would potentially identify unique or common mediating factors.

The results of this study suggest that the perioperative period could be exploited to control minimal residual disease by blocking primary tumor support for micro-metastases and by targeting pro-metastatic factors released by minimal residual disease, both before and after surgery. Specifically, identifying critical pro-metastatic factors secreted by the primary tumor in each patient, based on malignant tissue biopsy before surgery and/or in excised tumor following surgery, may enable perioperative use of an individually tailored combination of specific neutralizing antibodies. Analyses of serum samples before and after PT resection may complement this analysis. Neutralization of specific factors may also counteract the metastasis-promoting effects of surgery [1, 50], which may be partly mediated through stress- and inflammatory-induced excess release of pro-metastatic factors by the malignant tissue [51]. This may tilt the balance toward the eradication of metastases or arrest of their growth. The perioperative period harbors numerous risk factors for metastatic progression and was thus suggested to present a window of opportunity to exert a high impact on long-term cancer outcomes through various interventions [1, 31, 52].

## Conclusions

The herein findings may explain the rare but validated clinical phenomenon of spontaneous regression of recognized metastases following PT excision. The prevalence of post-operative regression of micro-metastases in cancer patients is currently unknown, due to technical limitations, but may occur in a substantial portion of patients. Factors secreted by the PT may play a critical role in enabling the initiation and progression of early-stage metastases, before such malignant foci become self-sufficient. Identifying and targeting specific factors may present a new therapeutic approach during the perioperative period.

## Methods

### Cancer model

A highly metastatic variant of the triple-negative breast adenocarcinoma cell line, MDA-MB-231^HM^ (a gift from Dr. Zhou Ou, Fudan University, Shanghai Cancer Center, China), was transduced with a codon-optimized firefly luciferase-mCherry vector as previously described [53]. Cells were cultured in Dulbecco’s modified Eagle’s medium (DMEM; Thermo Fisher Scientific) supplemented with 10% fetal bovine serum (FBS), 1% GlutaMAX (Thermo Fisher Scientific), 4.5 g/l d-glucose, and 110 mg/l sodium pyruvate. Cells were maintained at 37 °C and 5% CO_2_ and were mycoplasma-free.

### In vivo model of spontaneous metastasis

Mice were housed under SPF conditions on a 12-h dark/light cycle. Eight-week-old female BALB/c nu/nu mice (University of Adelaide, Australia, or Envigo, Israel) were injected with 2 × 10^5^ cells in 20 μl PBS into the fourth mammary fat pad (under 2% isoflurane anesthesia) to form a primary tumor. Primary tumors were measured by a caliper, and volume was calculated by the formula: (length × width^2^) × 0.5. Metastases were assessed by bioluminescence imaging using an IVIS spectrum apparatus (Perkin Elmer) following i.p. injection of 150 mg/kg d-luciferin sodium salt (Regis Technologies). Each animal was scanned twice, once for PT imaging (1–10 s exposure time) and once for metastases imaging (1 min exposure time) with the PT covered so the lower signal of the metastases will not be masked by the higher PT signal. Once the metastatic foci reached a total count of 10^6^ photons/s (~ 3–4 weeks post-injection), the primary tumor (average size of 80 mm^3^) was excised, and complete removal was verified by bioluminescence imaging. Follow-up of metastatic progression was conducted by bioluminescence imaging. At the end point, animals were euthanized 10 min following luciferin injection, and the lungs and lymph nodes were harvested for ex vivo imaging. All procedures were approved by Monash University or Tel-Aviv University Animal Ethics Committees.

### Surgical procedures

Primary tumor resection was performed under anesthesia with 2% isoflurane. A small incision in the skin was performed without injuring the peritoneal cavity to excise the PT. Following complete removal of the PT (which was verified by bioluminescence imaging), the skin was immediately sutured. For the sham surgery, an identical incision was made; however, the PT was untouched. The lesion was immediately sutured. Animals were randomly assigned to the experimental conditions.

### Tumor cell-conditioned medium preparation and use

CM was produced by incubating ~ 80% confluent MDA-MB-231^HM^ cells with serum-free medium for 24 h at 37 °C, 5% CO_2_. SM (i.e., serum-free medium, SM) was used as control. For in vivo supplementation of tumor secretome, CM was collected from ~ 30 million cells and filtered through a 0.45-μm strainer. CM (and the same volume of SM as control) was then concentrated by 3 kDa Amicon filters (Merck-Millipore) to a final volume of 100 μl/mouse and stored at − 20 °C until usage. On implantation day, Alzet osmotic mini-pumps (model 1003D) were loaded with either CM or SM and implanted i.p. immediately following tumor excision. Animals were randomly assigned to the experimental conditions. Based on an estimation that an excised primary tumor contains ~ 50 × 10^6^ secretome-producing cells (in ~ 80 mm^3^ primary tumor), we estimate that the amount of secretome factors released/day by the osmotic mini-pumps used is ~ 1/8 than the amount produced in vivo/day by the primary tumor.

### Apoptosis and adhesion studies

Cancer cells were cultured in growth media, washed, and seeded in very low numbers (~ 10% confluence, 50,000 cells/well in a 6-well plate in 2 ml media, 5260 cells/cm^2^) in either SM or CM to simulate low cell numbers in micro-metastases. For adhesion studies, cells were imaged for 8 h, in 20 min intervals, using the IncuCyte system (Essen Bioscience). The number of adhered cells was determined. For apoptosis studies, cells were incubated in CM or SM for 24 h, then washed and stained for AnnexinV-FITC (R&D systems) and 7AAD (R&D systems). Using flow cytometry, the percent of live/dead/early apoptotic cells was determined.

### Tube formation assay

Twenty-four-well plates wells were layered with 50-μl basement membrane extracellular matrix (Cultrex BME; Trevigen) and incubated for 30 min at 37 °C, 5% CO_2_. Then, human umbilical vein endothelial cells (HUVEC), reconstituted in either CM or SM, were seeded. Cells were imaged at 20 min intervals for 24 h using the IncuCyte system (Essen Bioscience). The average cell area (μm^2^) was analyzed by the IncuCyte software, and the number of tubes was counted.

### Human cytokine analysis

Human Cytokine Array (R&D systems, Proteome Profiler™ Array, ARY022) was used to compare relative expression levels of 102 soluble human proteins in CM samples and in plasma samples from (i) mice bearing a primary tumor, (ii) mice 1 day following primary tumor excision, and (iii) control mice. Each plasma sample was pooled from 3 mice. Analysis and quantification were used by Protein Array Analyzer in the ImageJ software.

### Enzyme-linked immunosorbent assay

MDA-MB-231^HM^ cells were seeded in serum-free medium (SM) in 6-well plates (10^6^ cells, 1.2 ml/well) for 24 h. The supernatant was collected, and levels of IL-8, MIF, Serpin E1, and PDGF-AA were assessed by human ELISA kits (R&D Systems) according to the manufacturer’s instructions. For each cytokine, an assessment was conducted twice in biological replications.

### Mass spectrometry-based secretome analysis

CM samples were centrifuged to eliminate cell debris followed by filtration and concentration using 3-kDa Amicon filters. The concentrated medium was mixed at a 1:1 ratio with 8 M urea and filtered again to reach a final volume of ~ 100 μl. Prior to protein digestion, proteins from the filtered samples were incubated with 1 mM dithiothreitol followed by 5 mM iodoacetamide. Proteins were digested overnight with LysC/Trypsin mix (Promega) and sequencing-grade modified Trypsin (Promega) at room temperature, followed by desalting and concentration on C_18_ StageTips [54]. Prior to MS analysis, peptides were eluted from StageTips using 80% acetonitrile, vacuum-concentrated, and diluted in MS loading buffer (2% acetonitrile, 0.1% formic acid). Liquid chromatography-mass spectrometry (LC-MS) was performed using the nano-ultra high-performance liquid chromatography system (UHPLC) (Easy-nLC1000, Thermo Fisher Scientific), followed by MS analysis on the Q-Exactive Plus mass spectrometer (Thermo Fisher Scientific). Peptides were separated by reverse-phase chromatography (50 cm long EASY-Spray PepMap columns; Thermo Fisher Scientific) with a 140-min linear gradient of water/acetonitrile. MS analysis was performed using a top 10 method in which every high-resolution MS scan was followed by fragmentation of the 10 most abundant peaks by higher-energy collisional dissociation (HCD).

### Proteomics data analysis

Mass spectrometry (MS) raw files were analyzed by MaxQuant [55] and the Label-free quantification algorithm [56]. MS/MS spectra were referenced to the Uniprot human proteome by the Andromeda search engine. A false discovery rate (FDR) of 0.01 was used on both the peptide and protein levels based on a decoy database. Statistical analysis was performed using the Perseus software [57], String database (www.string-db.org), and Cytoscape software. Enrichment analysis was performed relative to the identified secretome using Gene Ontology annotations from UniProt (Fisher exact test with an FDR threshold of 0.02).

### Neutralizing antibodies and their use

Mouse anti-human monoclonal antibodies (R&D Systems) were used to neutralize CXCL8/IL-8 (IgG1 Clone # 6217), Serpin E1/PAI-1 (IgG1 Clone # 242816), PDGF-AA (IgG1 Clone # 114506), and MIF (IgG1 Clone # 12302), and monoclonal mouse IgG1 served as isotype control (IgG1 Clone # 11711). For in vitro studies, all antibodies/isotype control (0.4 μg/100 μl) was added to either SM, 100% CM, or 50% CM (diluted with SM) for 1 h incubation at 37 °C. Cancer cells were cultured in growth media, washed, and incubated with antibodies/isotype control-containing media in 96-well plates (2500 cells, 100 μl/well). Proliferation was measured using Cell Proliferation Reagent WST-1 (Sigma-Aldrich) according to the manufacturer’s instructions (a minimum of quadruplicates per condition). Following 3, 6, and 21 h of incubation, WST-1 was added to each well for 2 h before absorbance intensity assessment. Each media preparation was also seeded with no cells, as a blank control condition, and its absorbance intensity was recorded and subtracted from matching media+cells reading. For in vivo supplementation, all antibodies/isotype control were injected once a day (1 μg each antibody/mouse, 4 μg isotype-control/mouse) for 2 days.

### Histology

To identify lung micro-metastases, the lungs were imaged ex vivo, and the lobes with bioluminescence-verified micro-metastases were collected, fixed in 4% paraformaldehyde, and paraffin-embedded. The lung sections (6 μm) were stained for hematoxylin and eosin (H&E). Metastatic foci were confirmed by a pathologist.

### Patient survival analysis

Using the METABRIC cohort [58] and TCGA provisional dataset (cBioPortal) [59, 60], we studied the patient outcomes in breast cancer and lung adenocarcinoma cohorts. For the breast cancer analysis, mRNA tumor expression levels of IL-8, PDGF-AA, Serpin E1, and MIF were divided into high and low groups by their median levels, omitting from the analysis patients with missing survival data. For the combined analysis of the four factors, high and low groups were formed based on the median of the mean of normalized expression levels of the four factors (mean signature level). Ten-year survival was chosen as the upper limit for follow-up, as longer periods may be more heavily contaminated by death from non-cancer-related causes. For the lung adenocarcinoma cohort, patients were stratified by their protein levels of IL-8, PDGF-AA, Serpin E1, and MIF, based on relative protein levels in RPPA assay. The survival curve was conducted according to the Kaplan-Meier analysis, and a hazard ratio was calculated in the R software.

### Statistical analysis

Repeated measures or factorial analysis of variance (ANOVA), with a pre-determined significance level of 0.05, was conducted. Provided significant group differences were found, Fisher’s protected least significant difference (Fisher’s PLSD) contrasts were performed to test pair-wise post hoc comparisons. Paired or unpaired Student’s *t* test was performed for comparing two experimental conditions (following *F*-test of equality of variance). All statistical tests were two-sided.

## Supplementary information

**Additional file 1: Fig. S1.** Excision of the primary tumor elicits gradual regression of early-stage metastases. **Table S1.** List of cytokines pointed out by the cytokine array, and criteria used for the selection process to suggest potential prominent factors. **Fig. S2.** ELISA validation of in-vitro tumor secretion of the chosen cytokines. **Fig. S3.** Elevated levels of Serpin E1, IL-8, MIF and PDGF-AA are correlated to poor survival in lung cancer patients. **Fig. S4.** Associations between levels of DKK1, IL-6, M-CSF and LIF and survival in breast cancer patients.

**Additional file 2: Table S2.** Extracellular proteins within the identified MDA-MB-231^HM^ secretome. **Table S3.** Identified proteins in MDA-MB-231^HM^ conditioned medium.

## Data Availability

The mass spectrometry proteomics data have been deposited to the ProteomeXchange Consortium [61] via the PRIDE partner repository [62] with the dataset identifier PXD008384 [63] and is available here http://www.ebi.ac.uk/pride/archive/projects/PXD008384. All other data is available in the main text or the supplementary materials.
