# Publisher Correction: Spontaneous regression of micro-metastases following primary tumor excision: a critical role for primary tumor secretome

**DOI:** 10.1186/s12915-020-00932-y

**Published:** 2020-12-16

**Authors:** Lee Shaashua, Anabel Eckerling, Boaz Israeli, Gali Yanovich, Ella Rosenne, Suzana Fichman-Horn, Ido Ben Zvi, Liat Sorski, Rita Haldar, Ronit Satchi-Fainaro, Tamar Geiger, Erica K. Sloan, Shamgar Ben-Eliyahu

**Affiliations:** 1 Sagol School of Neuroscience and School of Psychological Sciences, Tel Aviv University, 69978 Tel Aviv, Israel; 2 Department of Human Molecular Genetics and Biochemistry, Sackler Faculty of Medicine, Tel Aviv University, Tel Aviv, Israel; 3 Pathology Institute, Rabin Medical Center, Tel Aviv University, Petach Tikva, Israel; 4 Neurosurgery Department, Rabin Medical Center, Tel Aviv University, Petach Tikva, Israel; 5 Department of Physiology and Pharmacology, Sackler Faculty of Medicine, Tel Aviv University, Tel Aviv, Israel; 6 Drug Discovery Biology Theme, Monash Institute of Pharmaceutical Sciences, Monash University, Parkville, VIC 3052 Australia

**Correction to: BMC Biol 18, 163 (2020)**

**https://doi.org/10.1186/s12915-020-00893-2**

The original publication of this article [1] contained an incorrect version of figure 4, and an incorrect version of figure S4 in Additional file 1. The wrong version of both files, which contained an incorrect color legend, was accidentally used during typesetting of the article.

In this correction article the correct and incorrect version of figure 4 are published along with the updated Additional file 1.

The original article has been updated.

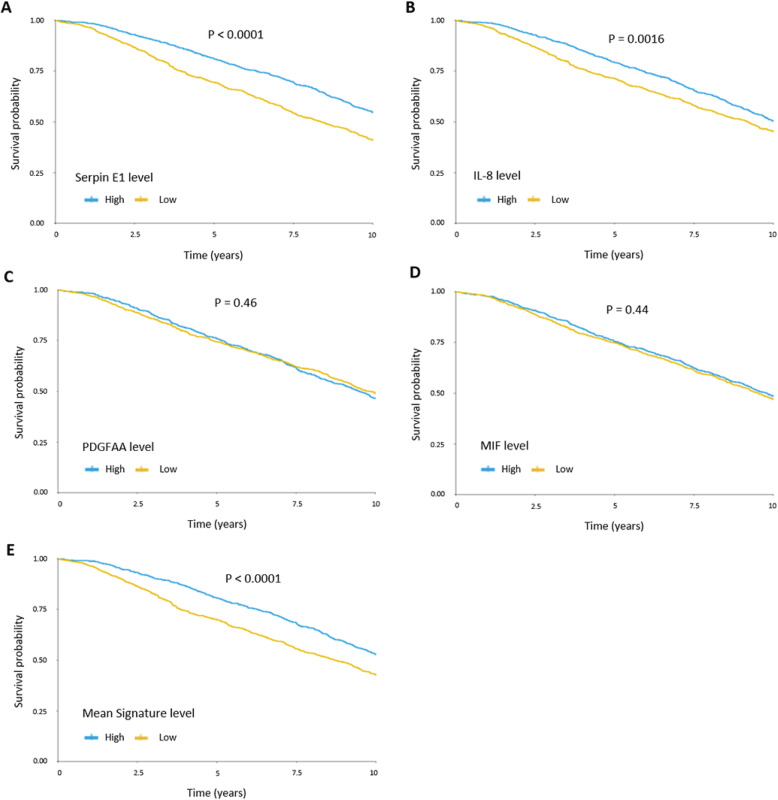



**Incorrect figure 4.**

**Correct** Figure 4.

**Fig. 4:**
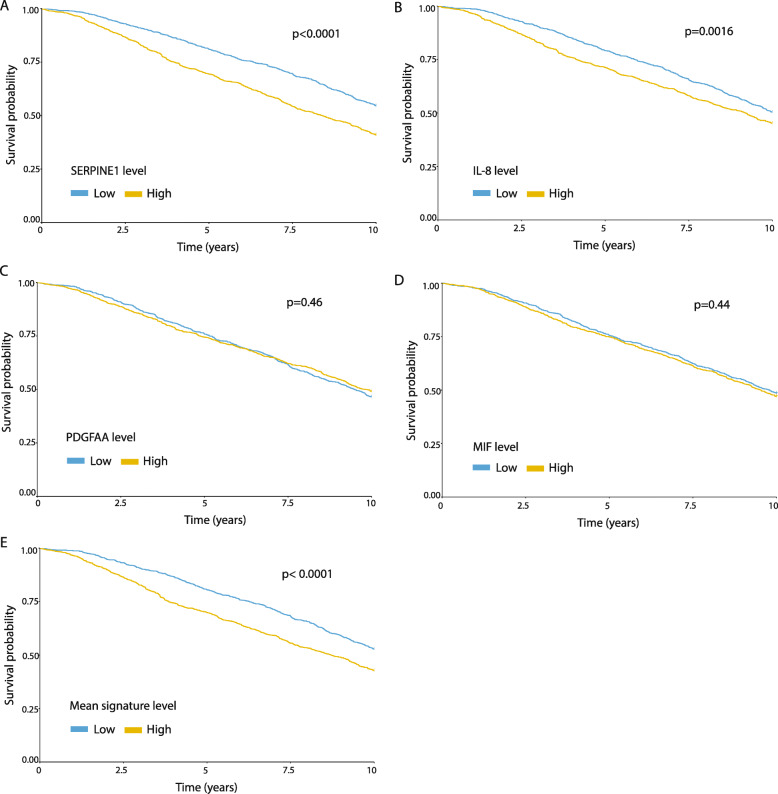
Associations between levels of Serpin E1, IL-8, MIF, and PDGF-AA and survival in breast cancer patients. The METABRIC dataset was used to assess the association between expression levels of Serpin E1 (**a**), IL-8 (**b**), PDGF-AA (**c**), MIF (**d**) and the mean signature levels of all 4 factors (median of the mean of normalized expression levels of the four factors) (**e**), with 10-year survival. Protein levels were classified as higher or lower than the median, and the association to 10-year survival was assessed by the Kaplan-Meier analysis (*n* = 952 per group). *p* value was calculated using two-sided log rank test

## Supplementary Information

**Additional file 1: Fig. S1.** Excision of the primary tumor elicits gradual regression of early-stage metastases. **Table S1.** Cytokines pointed out by the cytokine array. **Fig. S2.** ELISA validation of in-vitro tumor secretion of the chosen cytokines. **Fig. S3.** Elevated levels of Serpin E1, IL-8, MIF and PDGF-AA are correlated to poor survival in lung cancer patients. **Fig. S4.** Associations between levels of DKK1, IL-6, M-CSF and LIF and survival in breast cancer patients.
