# Supplementary material for: Spontaneous regression of micro-metastases following primary tumor excision: a critical role for primary tumor secretome
Source: BMC Biol. 2020 Nov 6;18:163. doi: 10.1186/s12915-020-00893-2 (PMC7646068; doi:10.1186/s12915-020-00893-2)
Supplement: Supplementary file 1 — Additional file 1: Fig. S1. Excision of the primary tumor elicits gradual regression of early-stage metastases. Table S1. List of cytokines pointed out by the cytokine array, and criteria used for the selection process to suggest potential prominent factors. Fig. S2. ELISA validation of in-vitro tumor secretion of the chosen cytokines. Fig. S3. Elevated levels of Serpin E1, IL-8, MIF and PDGF-AA are correlated to poor survival in lung cancer patients. Fig. S4. Associations between levels of DKK1, IL-6, M-CSF and LIF and survival in breast cancer patients. [file 12915_2020_893_MOESM1_ESM.docx]

**Supplementary Materials**


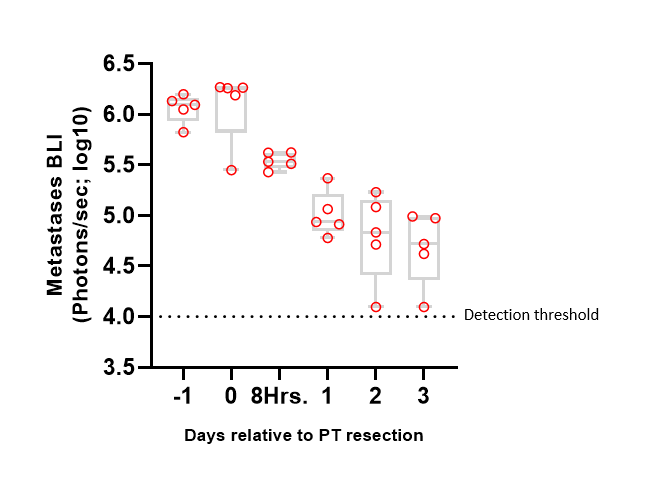


**Figure S1: Excision of the primary tumor elicits gradual regression of early-stage metastases.** In vivo quantification of lung and lymph node metastases by bioluminescence imaging (BLI) 1 day before, immediately before (day 0), 8 hours and 1 to 3 days after primary tumor (PT) resection (n=5). Whiskers represent min and max points.

Table S1 – Cytokines pointed out by the cytokine array

| CM *in vitro* secretion  (normalized mean intensity above 6,000) | Plasma levels of tumor bearing mice (normalized mean intensity) | Ratio of plasma levels from tumor-bearing/control mice | Ratio of plasma levels from tumor- bearing/excised tumor mice |
| --- | --- | --- | --- |
| Serpin E1 | 4786 (+) | 2.01 (+) | 1.43 (+) |
| PDGF-AA | 38487 (+) | 2.10 (+) | 1.31 (+) |
| IL-8 | 2683 (+) | 1.81 (+) | 1.45 (+) |
| MIF | 3195 (+) | 2.47 (+) | 1.73 (+) |
| DKK1 | 3984 (+) | 3.21 (+) | 1.53 (+) |
| MCP-1 | 2777 (+) | 1.51 (+) | 1.22 |
| Thrombospondin-1 | 2474 (+) | 2.41 (+) | 1.06 |
| uPAR | 2139 (+) | 1.73 (+) | 1.14 |
| IL-22 | 2037 (+) | 1.52 (+) | 0.90 |
| Pentraxin-3 | 4863 (+) | 1.34 | 1.21 |
| FGF-19 | 2277 (+) | 0.97 | 0.57 |
| IL-17A | 4287 (+) | 0.69 | 0.88 |
| M-CSF | 2849 (+) | 1.43 | 1.30 (+) |
| IL-11 | 3812 (+) | 1.32 | 1.32 (+) |
| IL-6 | 3727 (+) | 1.22 | 1.39 (+) |
| Osteopontin | 2921 (+) | 1.06 | 0.91 |
| Angiopoietin-2 | 2734 (+) | 0.94 | 0.76 |
| Vitamin D | 3613 (+) | 1.17 | 1.27 |
| SDF-1 | 5751 (+) | 0.91 | 0.75 |
| FGF | 2605 (+) | 0.95 | 1.02 |
| LIF | 1854 | 2.05 (+) | 1.28 |
| Angiogenin | 950 | 1.41 | 0.86 |
| EMMPRIN | 1627 | 0.41 | 0.59 |
| GDF-15 | 1408 | 0.89 | 0.67 |
| VEGF | 1148 | 0.78 | 0.89 |
| IGFBP-3 | 1358 | 1.22 | 0.85 |
| Cystatin C | 1335 | 1.23 | 0.74 |
| GM-CSF | 1322 | 0.42 | 0.54 |

**Table S1: List of cytokines pointed out by the cytokine array, and criteria used for the selection process to suggest potential prominent factors.** The left column represents the 28 upregulated cytokines in *in vitro* conditioned medium of MDA-MB-231^HM^ (criterion for upregulation: intensity>6000). Out of these, 20 cytokines were upregulated in plasma of tumor-bearing mice (criterion for upregulation: intensity>2000). Then we selected for cytokines which their levels in tumor bearing mice were upregulated compared to both control mice (criterion for upregulation: ratio > 1.5) and mice a day following tumor resection (criterion for upregulation: ratio > 1.3). Selected cytokines were those which passed all four criteria. (+) represents criteria positive.

**Figure S2: ELISA validation of *in-vitro* tumor secretion of the chosen cytokines.** ELISA assessment of the levels of IL-8, MIF, Serpin E1 and PDGF-AA in the supernatant of MDA-MB-231^HM^ cells. Cells were seeded in serum-free medium (SM) for 24hrs, the supernatant was collected and assessed by human ELISA kits (one for each cytokine). Assessment was conducted twice in biological replications for each cytokine. Error bars represent mean $\pm$ SE


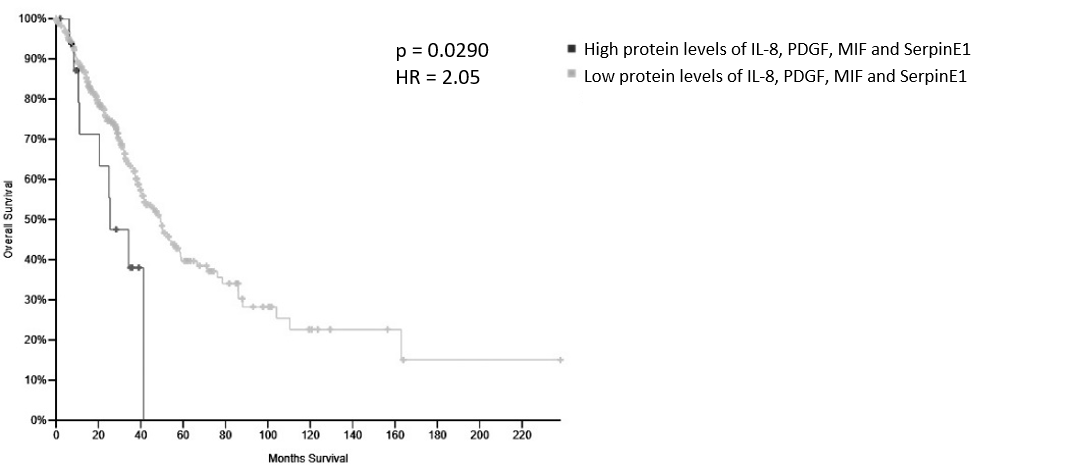


**Figure S3: Elevated levels of Serpin E1, IL-8, MIF and PDGF-AA are correlated to poor survival in lung cancer patients.** Kaplan-Meier analysis of patients stratified by high/low proteins levels of IL-8, MIF, PDGF-AA, and Serpin E1 (n=18 in high protein levels, and n= 338 in low protein levels).


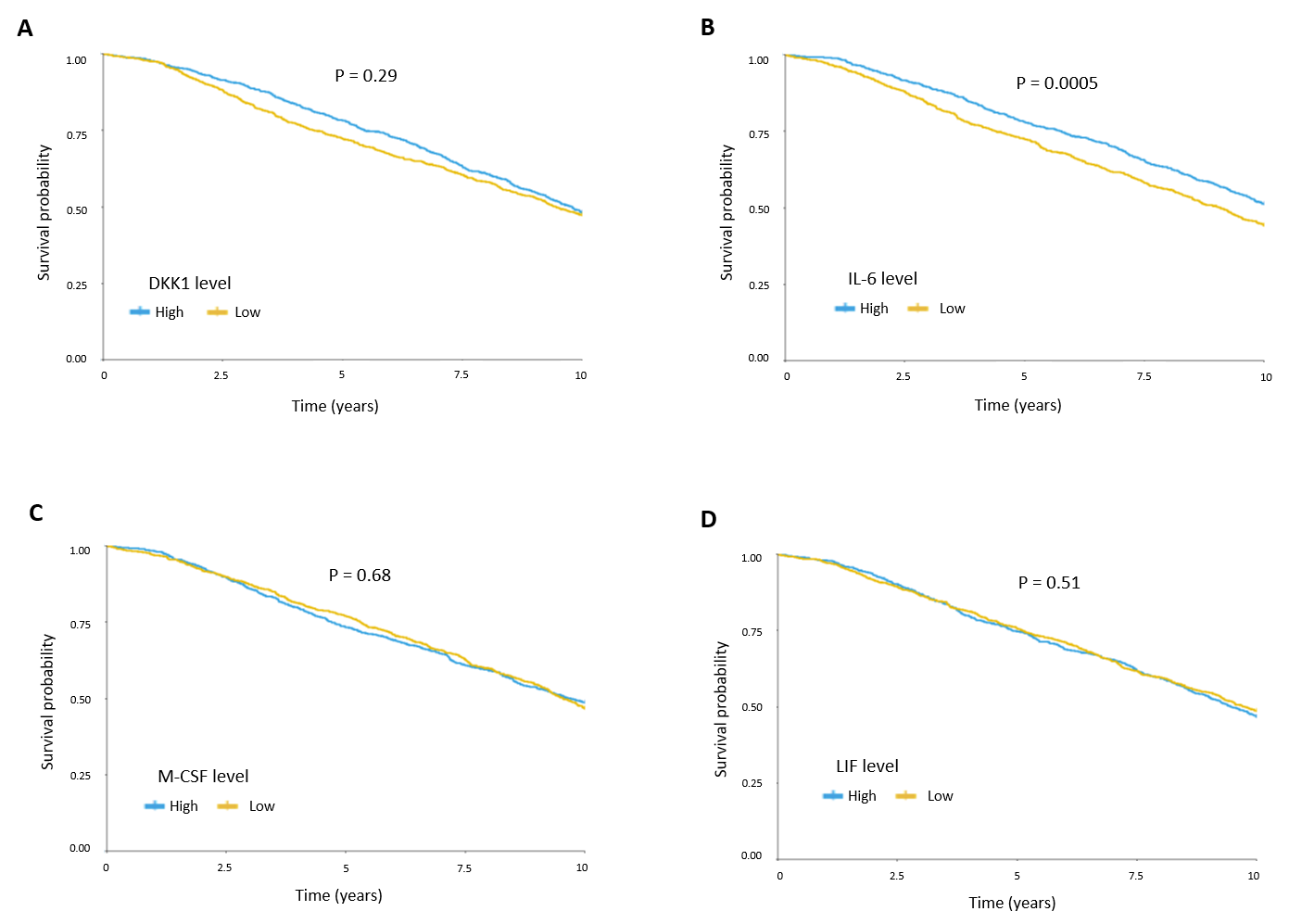


**Figure S4: Associations between levels of DKK1, IL-6, M-CSF and LIF and survival in breast cancer patients.** The METABRIC dataset was used to assess the association between expression levels of DKK1 **(A)**, IL-6 **(B)**, M-CSF **(C)**, and LIF **(D),** with 10-year survival. Protein levels were classified as higher or lower than the median, and the association to 10-year survival was assessed by Kaplan Meier Analysis (n=952 per group). P-value was calculated using two-sided log rank test.
